# Source-Sink Mismatch Causing Functional Conduction Block in Re-Entrant Ventricular Tachycardia

**DOI:** 10.1016/j.jacep.2017.08.019

**Published:** 2018-01

**Authors:** Edward J. Ciaccio, James Coromilas, Andrew L. Wit, Nicholas S. Peters, Hasan Garan

**Affiliations:** a Department of Medicine, Division of Cardiology, Columbia University College of Physicians and Surgeons, New York, New York; b ElectroCardioMaths Programme, Imperial Centre for Cardiac Engineering, Imperial College London, London, United Kingdom; c Department of Medicine, Division of Cardiovascular Disease and Hypertension, Rutgers University, New Brunswick, New Jersey; d Department of Pharmacology, Columbia University College of Physicians and Surgeons, New York, New York

**Keywords:** activation, MRI, re-entry, ventricular tachycardia, wavefront, IBZ, infarct border zone, VT, ventricular tachycardia

## Abstract

Ventricular tachycardia (VT) caused by a re-entrant circuit is a life-threatening arrhythmia that at present cannot always be treated adequately. A realistic model of re-entry would be helpful to accurately guide catheter ablation for interruption of the circuit. In this review, models of electrical activation wavefront propagation during onset and maintenance of re-entrant VT are discussed. In particular, the relationship between activation mapping and maps of transition in infarct border zone thickness, which results in source-sink mismatch, is considered in detail and supplemented with additional data. Based on source-sink mismatch, the re-entry isthmus can be modeled from its boundary properties. Isthmus boundary segments with large transitions in infarct border zone thickness have large source-sink mismatch, and functional block forms there during VT. These alternate with segments having lesser thickness change and therefore lesser source-sink mismatch, which act as gaps, or entrance and exit points, to the isthmus during VT. Besides post-infarction substrates, the source-sink model is likely applicable to other types of volumetric changes in the myocardial conducting medium, such as when there is presence of fibrosis or dissociation of muscle fibers.

Ventricular tachycardia (VT) is a life-threatening arrhythmia and major clinical problem. Although interest in research to develop improved methods of treatment has somewhat waned in recent years as compared with atrial fibrillation research (1), the problem of efficacious treatment of VT in the electrophysiology laboratory has not yet been solved. Often, a re-entrant circuit is the source of clinical VT in patients with healing or healed myocardial infarction (2). If the arrhythmia is refractory to antiarrhythmic drugs, the patient will often be treated with catheter ablation. The ablation catheter delivers radiofrequency energy to the presumed location or locations of arrhythmogenicity to prevent arrhythmia recurrence (3). Usually, in clinical cases, these areas are located on the endocardial surface or intramurally, although they can also be transmural or entirely epicardial in origin (4). In the electrophysiology laboratory, the goal is to determine the arrhythmogenic region and to ablate it to prevent electrical conduction there and thereby to stop re-entrant VT from recurring. To do this efficaciously, the mechanism of re-entrant VT should be known. Although several mechanisms have been proposed, there is no general acceptance of a model that describes the electrophysiological events leading to the onset and maintenance of re-entrant VT. In this review, proposed mechanisms of re-entrant circuit formation and maintenance are compared and contrasted. Emphasis is given to the source-sink (current-load) model, in which localized volumetric changes in the conducting medium cause the activation wavefront to accelerate or decelerate, depending upon the availability of electrical current for distal activation. The work is complementary to that recently published elsewhere (5). In the prior work, a derivation of wavefront curvature equations describing source-sink mismatch was given and origins of source-sink mismatch were defined. Herein, the shape of the superficial infarct region (i.e., scar tissue) with respect to the boundary characteristics of the re-entrant VT isthmus, and to optimal catheter ablation locations for preventing VT reinduction, is reviewed. Additional data are included to supplement the reviewed material.

A re-entrant circuit driving VT typically consists of the components shown in the diagram of Figure 1, and has a double-loop configuration. The electrical activation wavefront propagates through viable myocardial tissue comprising the infarct border zone (IBZ), which is a constrained area of the myocardial conducting medium that is bounded by infarct in proximity and often by the heart surface. The wavefront travels as a single impulse through a region known as the isthmus of the re-entrant circuit, also known as the diastolic pathway, inner pathway, and central common pathway (6). Functional or fixed areas of conduction block prevent electrical conduction laterally outward from the isthmus, as noted by the thick lines or surfaces. At the isthmus exit, the impulse bifurcates, and travels around as 2 distinct wavefronts in the opposite direction. This region outside the isthmus is known as the outer pathway (7). At the isthmus entrance, the wavefronts coalesce, and then travel again through the isthmus region as a single electrical impulse.

**Figure 1:**
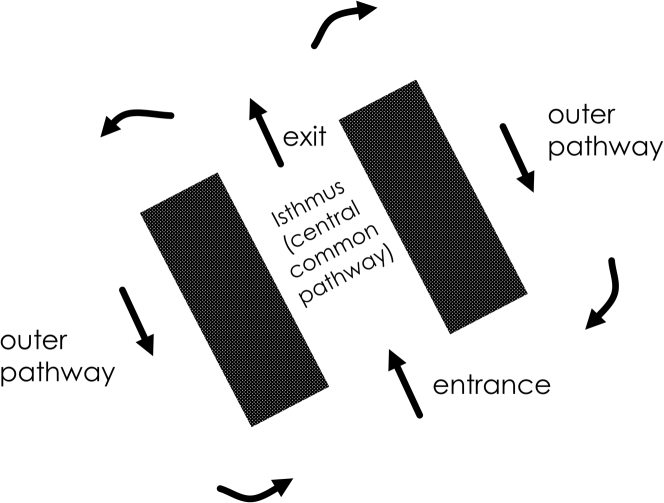
Diagram of a Double-Loop Re-Entrant Circuit **Dark panels** show areas that do not conduct electrically. **Arrows** denote the direction of propagation of the electrically activating wavefront.

A radiofrequency ablation lesion across the isthmus, where the impulse is constrained, would be most effective to prevent recurrence of re-entrant VT; however, finding this region with a roving ablation catheter can be problematic (8). First, the particular re-entrant circuit morphology responsible for clinical VT may not be inducible by programmed electrical stimulation, or it may not be well-tolerated by the patient, meaning that it must be terminated before being completely mapped. Second, current mapping techniques are not always entirely accurate. Voltage mapping seeks areas of very low voltage to be used as candidate arrhythmogenic regions 9, 10. The method of concealed entrainment seeks to pace within the circuit by electrical stimulation during VT, with a cycle length equal to the VT cycle length (2). The location where the activation wavefront of the re-entrant circuit is advanced in time, but the signal shapes of the 12-lead electrocardiogram are otherwise unaffected, is sought for catheter ablation. This spot tends to reside at the exit of the re-entry isthmus. Pace-mapping can also be done during sinus rhythm, using a cycle length similar to the cycle length of the clinical re-entrant tachycardia (11). However, all of these methods suffer from limited accuracy for localizing the best site and the best orientation of the lesion to create by catheter ablation. Thus, the circuit may be incompletely interrupted, raising the possibility of VT recurrence and the need for follow-up procedures, and morbidity when ablated areas interfere with normal heart function. Mapping also requires significant procedure time (12), increasing the amount of radiation the patient receives from fluoroscopy, and increasing the cost of the procedure.

## Models of Post-Infarction Re-Entrant VT

**Figure 2:**
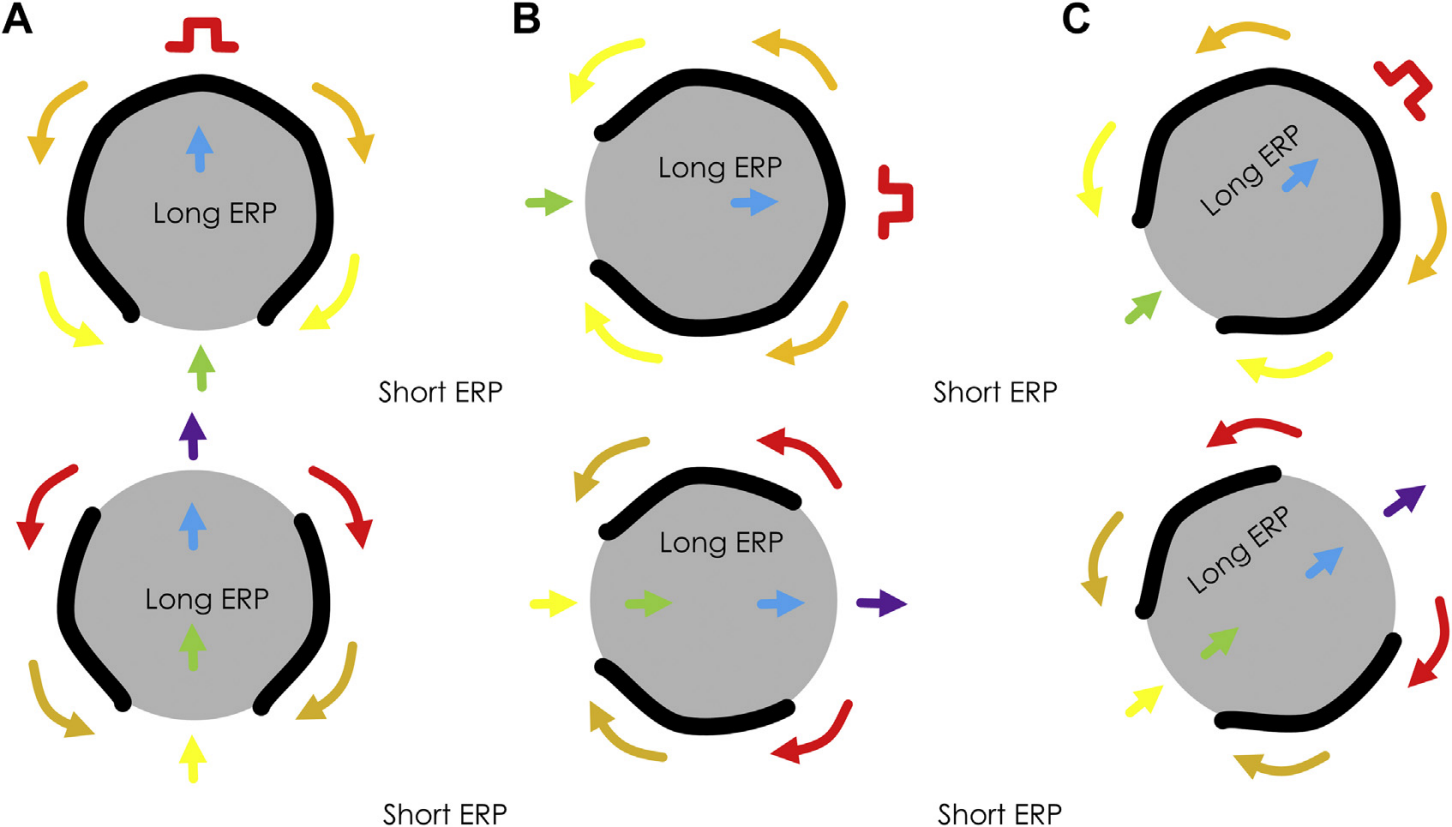
Refractory Model of Re-Entrant Ventricular Tachycardia **Arrows from red to violet** denote earlier to later activation, respectively. The stimulus site is noted by the **red symbol**. **(Top)** Premature stimulation. **(Bottom)** Re-entrant ventricular tachycardia forming after premature excitation. **AtoC** show 3 examples of re-entrant ventricular tachycardia induction based upon the refractory model. ERP = effective refractory period.

For improved detection of optimal ablation sites to interrupt the re-entrant circuit, it is important to develop a model that accurately reflects the underlying electrophysiologic phenomena by which re-entrant VT is initiated and maintained. Such a model could then be used to improve the explanation of the observed phenomena, and to plan the best strategy for rapid and accurate localization of arrhythmogenic regions prior to catheter ablation. Various models have been proposed over the years to describe how re-entrant circuits form and are maintained. In Figure 2, 1 such model is shown, based on refractoriness (13). Suppose the substrate consists of islands with long refractory periods and surrounding regions with shorter refractory period as depicted (Figure 2A). Further suppose that the region is being electrically paced with S1-S1 stimuli from the point shown by the pulse symbol. Let an S1-S2 stimulus pulse with short coupling interval then be delivered. This premature stimulus wavefront travels to the refractory region, whereupon it blocks functionally because of refractoriness. The activation wavefront then bifurcates and travels around the refractory region. If recovery of excitability occurs prior to the distinct wavefronts propagate beyond the distal end of the refractory region, they can then enter it as shown (green arrow). Electrical activity thereupon proceeds through the formerly refractory region in the opposite direction. Supposing that recovery of excitability has occurred in proximity to the stimulus site, the impulse can then re-enter the previously excited region so that re-entry is initiated. The impulse bifurcates and forms a double-loop re-entrant circuit (lower drawing, Figure 2A). If the stimulus site is changed, the same basic circuit morphology will occur, as shown in Figures 2B and 2C, although some alterations in the timing and shape of the isthmus may result from anisotropic conduction (i.e., wavefront propagation that is slowed transverse to muscle fibers because of differences in gap junctional connections, as compared with longitudinal wavefront propagation). Based on the refractory mechanism of Figure 2, the number of possible re-entry morphologies is multitudinous, depending upon the programmed stimulus location. Induction of any particular morphology depends highly on stimulus site location. There may also be instability and polymorphic tachycardia resulting from spatial inhomogeneities in refractoriness (14). Undoubtedly, this model is valid and a likely cause for some cases of ischemic and nonischemic tachycardia. Often however, 1 or at most 2 or 3 re-entrant circuit morphologies are observed from any location driving ventricular tachycardia in both canine models and clinical studies 15, 16; thus, it would not seem likely that this model would be valid in all cases.

**Figure 3:**
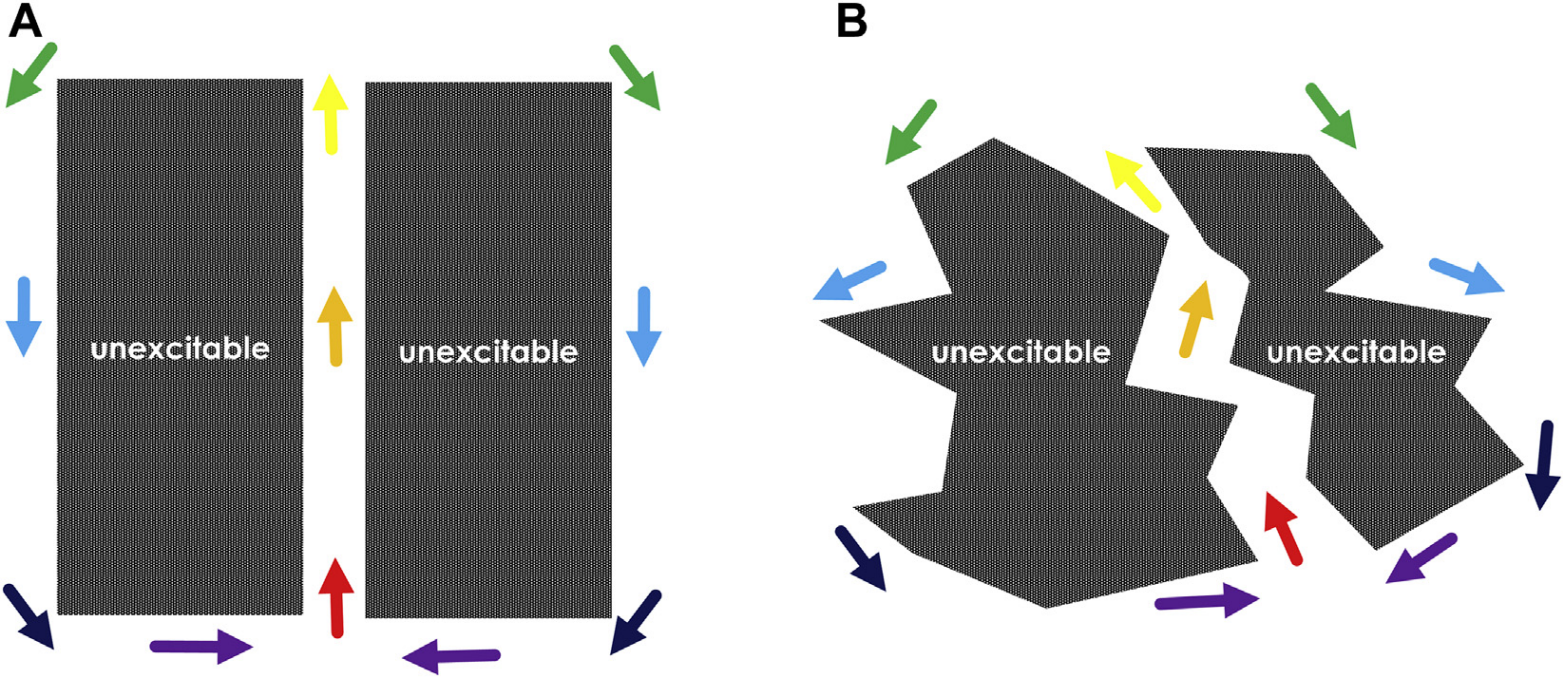
Fixed Obstacle Model of Re-Entrant Ventricular Tachycardia **Dark areas** correspond to fixed obstacles. **Colored arrows from red to violet** denote earlier to later activation. Channels through which conduction occurs are **white areas** between the unexcitable obstacles **(arrows from red to yellow)**. Propagation of the activation wavefront also occurs along the outer circuit pathway **(arrows from green to violet)**. **AandB** show examples of the fixed obstacle model.

Another possibility to explain the mechanism of re-entrant ventricular tachycardia is depicted in Figure 3. Fixed areas of unexcitable tissue are shown. In Figure 3A, a straight channel structure of conducting medium exists between the unexcitable areas, as has been observed in a porcine model (17). In Figure 3B, the channel has a zigzag structure 18, 19. Suppose an electrical impulse enters the channel depicted in Figures 3A or 3B from 1 end (red arrow in each panel). It travels through the isthmus region, exits, bifurcates, and then propagates as 2 wavefronts in the opposite direction along the outer pathway. The distinct wavefronts then coalesce (violet arrows) and re-enter the isthmus as a single impulse. In both cases, classic double-loop re-entrant circuits are formed. However, as with the refractory model, these models are also likely not valid in all cases. First, they do not elucidate how a premature excitation wavefront results in the formation of a unidirectional block line leading to re-entry. Second, large, fixed unexcitable regions are usually not observed in either canine or clinical post-infarction. Oftentimes rather, there is activation throughout the entire arrhythmogenic region during sinus rhythm (20). Third, during re-entrant VT, conduction block often occurs along thin lines bounding the isthmus laterally, not over large surface areas as shown in Figure 3.

The models for VT circuit mechanism depicted in Figures 2 and 3 are both based upon inhomogeneous surface areas. In the following sections, the source-sink (current-load) mismatch model, which can be used to explain the observed phenomena leading to onset and maintenance of re-entrant VT, is reviewed and elucidated. For this latter model, straight or curved line boundaries, rather than surface areas, are responsible for conduction block leading to and maintaining re-entry.

## Wavefront Curvature at Areas of Source-Sink Mismatch

**Figure 4:**
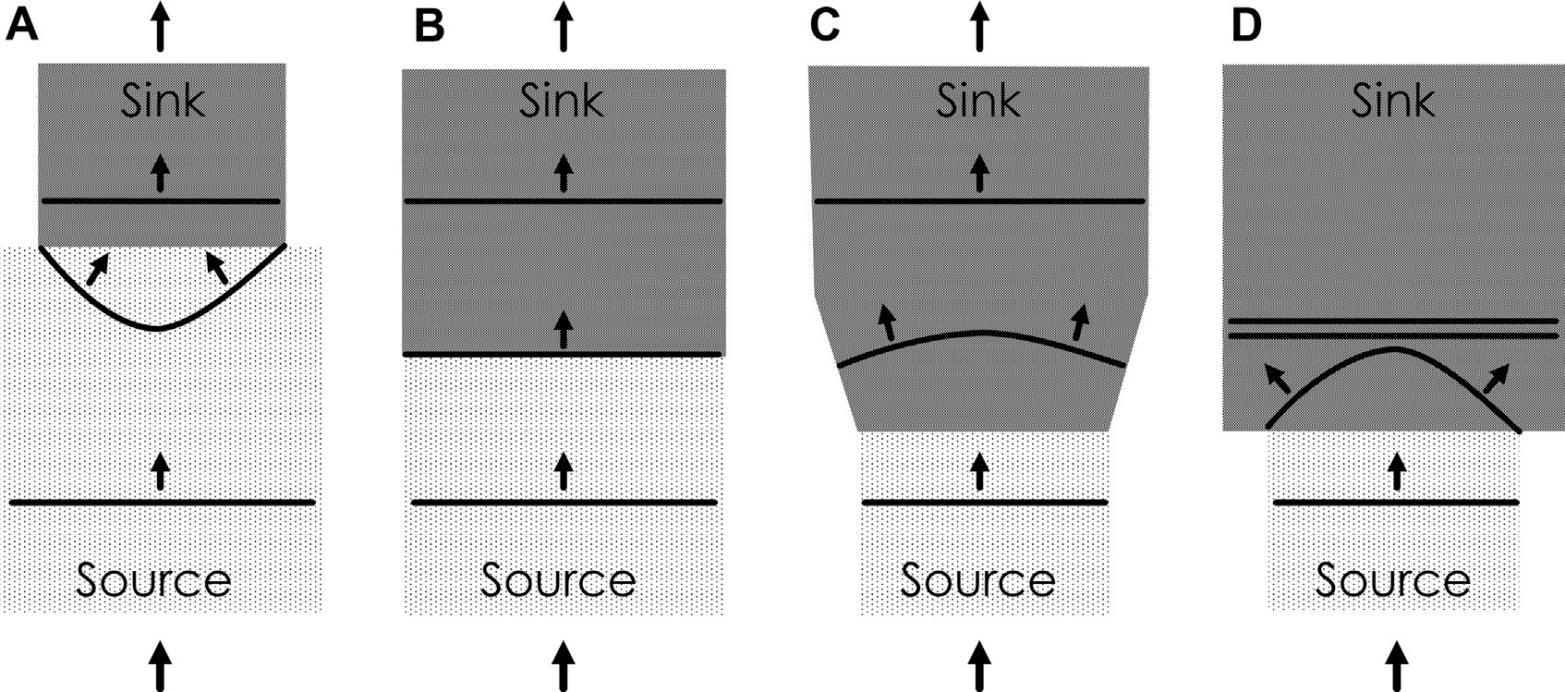
Source-Sink Mismatch and its Effect on Electrical Conduction at the Isthmus Boundary When the source volume is larger **(A)** or the same **(B)** compared with the sink volume, activation proceeds because there is sufficient electrical current to activate myocardial cells in the distal direction. When the source volume is smaller than the sink volume, however, the activation wavefront slows **(C)** or blocks **(D)** (the latter phenomenon is denoted by the **double black line**) because there is less electric current available to activate myocardial cells in the distal direction.

Source-sink mismatch alters the conduction velocity at boundaries between inhomogeneous tissue volumes 21, 22, 23, 24, 25. Shown in Figure 4, the activation wavefront propagates though a volume of tissue, the source (light patterned gray color). If in the travel direction, the subsequent localized volume of conducting tissue, the sink, is smaller as compared with the source (Figure 4A), propagation of the activation wavefront will proceed; there is sufficient electrical current available to conduct in the distal direction, and the wavefront actually accelerates. When the source and sink are the same size (Figure 4B), the wavefront propagates with no change in speed. However, suppose that the activation wavefront propagates from a source region with relatively small volume. If the tissue going forward, the sink, is substantially larger in volume, the wavefront will slow (Figure 4C) or even block functionally (double black lines, Figure 4D) because of the insufficiency of available current from the source to activate the larger-volume sink 21, 22, 23, 24, 25. The wavefront shape depends upon the available current versus load: it is concave and speeds up when the distal volume conductor is lesser in size (Figure 4A), it is a straight line (rectilinear) when there is no change in the geometry of the conducting medium in the forward direction (Figure 4B), and it becomes convex with slowing or block when the distal volume conductor is greater in size (Figures 4C and 4D, respectively). Boundary differences such as those depicted in Figure 4 are caused by spatial differences in the geometry of the conducting medium, as can occur in post-infarction (26), but also by fibrosis, as has been shown to occur in atrial tissue (27), and by discontinuities in muscle fiber bundles (28).

## Source-Sink Model Equations

**Figure 5:**
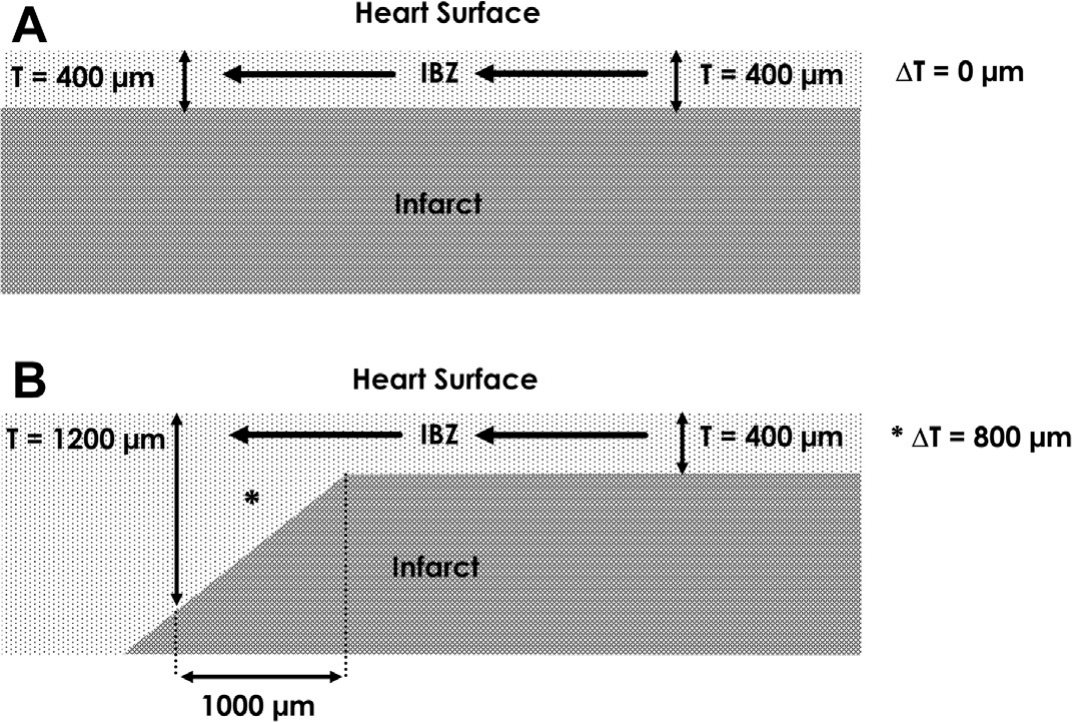
Configuration for Source-Sink Mismatch in the IBZ Activation wavefront propagates in the IBZ, superficial to the infarct region, from right to left as noted by the **arrows**. **(A)** No change in thickness T. **(B)** Change from thinner to thicker tissue, which can result in functional conduction block for the geometric dimensions shown. ΔT = the spatial change in thickness of the conducting medium per unit distance; IBZ = infarct border zone.

An equation that can be used to describe the conduction velocity of the activation wavefront at areas of source-sink mismatch is 5, 26:

(Equation 1) $$\theta =\theta_{o}-D\;\frac{\Delta T}{c\;\cdot \;T}$$

where *θ* is the conduction velocity of the propagating wavefront; *θ*_*o*_ is the conduction velocity that the propagating wavefront would have if there were no source-sink mismatch, and it can be approximated as a constant with value of 0.4 mm/ms (5); *D* is the diffusion coefficient, which can be approximated as a constant with value 0.2 mm^2^/ms (29); *T* is the thickness of the conducting medium; and *ΔT* is the spatial change in thickness of the conducting medium per unit distance (i.e., space step *c*) (26). Thus, to estimate *θ*, only thickness *T* requires measurement, whereas *ΔT* is calculated and *D* and *θ*_*o*_ are approximated as constants. An example is shown in Figure 5. For the configuration of panel A, there is no change in IBZ thickness *T* (*ΔT* = 0) and therefore no change in wavefront curvature; the leading edge is rectilinear. The wavefront conducts from right to left along the light gray IBZ region, superficial to the nonconducting darker gray infarct area. Thus, for the configuration shown in Figure 5A:

$$\theta =\theta_{o}-D\;\frac{\Delta T}{c\;\cdot \;T}$$

$$\theta =\theta_{o}-D\;\frac{0}{c\;\cdot \;T}$$

(Equation 2) $$\theta =\theta_{o}=0.4\;\text{mm/ms}$$

In Figure 5B, however, there is a change from thin to thicker IBZ in the propagation direction (right to left). Over an interval of *c* = 1 mm (1,000 μm), the thickness changes from *T* = 400 μm to *T* = 1,200 μm. Therefore:

$$\theta =\theta_{o}-D\;\frac{\Delta T}{c\;\cdot \;T}$$

$$\theta =0.4\frac{mm}{ms}-0.2\frac{mm^{2}}{ms}\;\frac{1.2\;mm-0.4\;mm}{1.0\;mm\;\cdot \;0.4\;mm}$$

(Equation 3) $$\theta =0\frac{mm}{ms}$$

Hence, for the configuration depicted in Figure 5B, the wavefront curvature becomes critically convex and the conduction velocity goes to zero. Thus, *ΔT/T* for conduction block to occur is ∼ 2 when the space step *c* = 1 mm. By calculating *θ* based on the geometry of the conducting medium, it is therefore possible to predict where functional block will occur (i.e., at locations where *θ* approaches or reaches a value of zero [*θ → 0*]). This will transpire when the right-hand term in the equation is approximately as large or even larger than *θ*_*o*_ and thus where *ΔT* is large and *T* is small in magnitude. In canine post-infarction, the re-entry isthmus location is the thinnest region of surviving IBZ, it tends to be located subepicardially 26, 30, 31, and has an average thickness *T* = 231 μm (0.23 mm), with a much greater average thickness *T* = 1440 μm (1.44 mm) in the outer pathway (26). Thus, at the boundaries of isthmus to outer pathway, the sharp changes from very thin to thicker tissue would be in agreement with Figure 5B and meet the criteria for functional block to occur.

Of note, the ability of an activation wavefront to propagate through an isthmus of a given thinness is also dependent upon the frequency of stimulation 23, 32. Typically, functional conduction block resulting from source-sink mismatch can occur as the wavefront propagates outwardly when the isthmus thickness *T* ≤ 500 μm 5, 32, 33, *ΔT* per unit space step *c* is large, and with the time between oncoming activation wavefronts in the range of VT cycle length intervals, approximately 175 to 300 ms (34). At the short coupling intervals for S1-S2 typically encountered during programmed electrical stimulation, on the order of 125 to 165 ms, however, functional conduction block can also occur at locations where *ΔT/T* is lesser in magnitude (32). Whereas, at sinus rhythm coupling intervals, approximately 350 ms or greater, functional block may not occur even at locations where *ΔT/T* is very large.

## The Isthmus Boundary Based Upon Source-Sink Model

**Figure 6:**
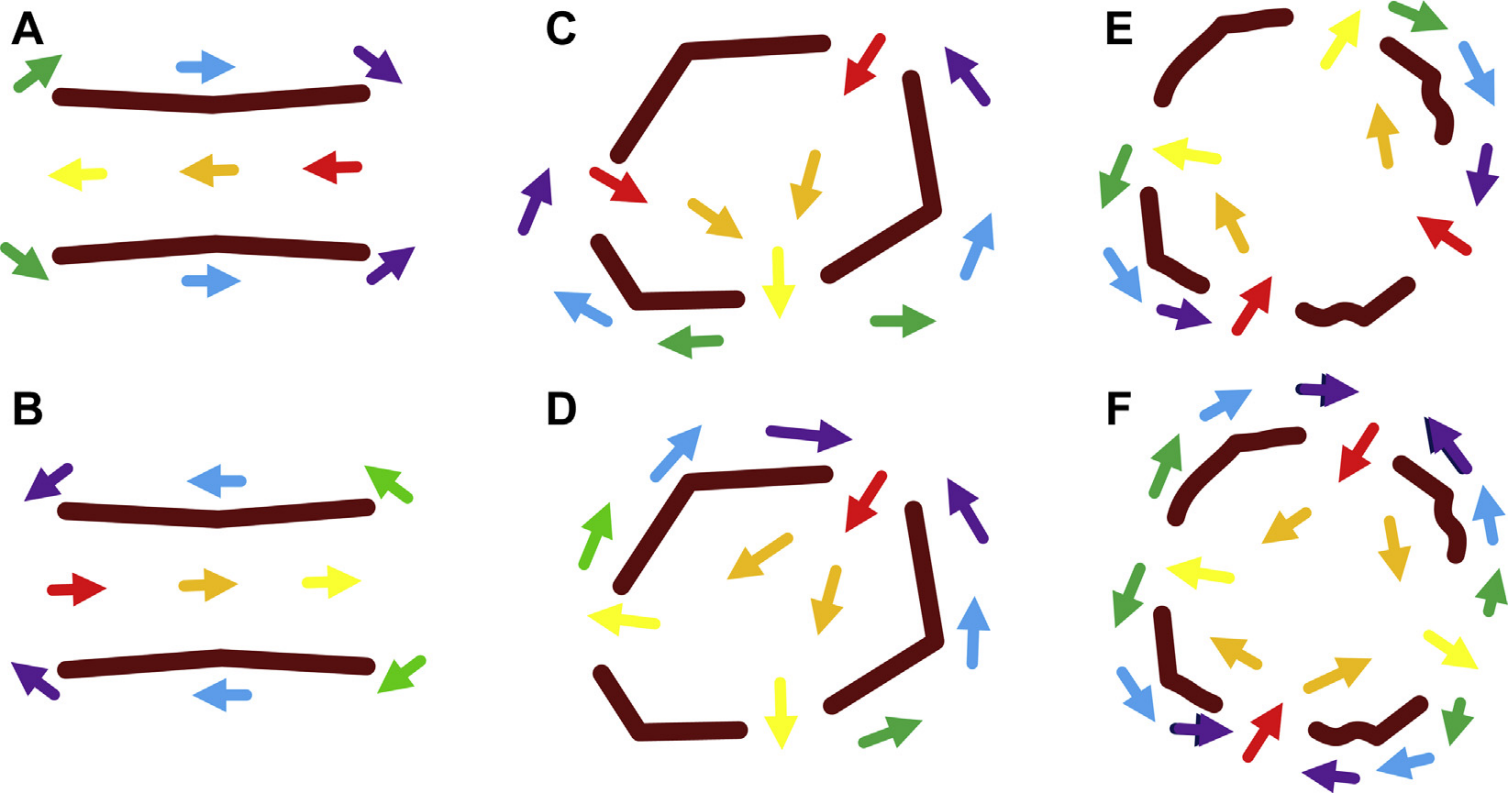
Isthmus Boundary in Re-Entrant Ventricular Tachycardia **(A, B) Two block lines** and **2 gaps**. The overall isthmus boundary has a rectangular shape. **Red to violet arrows** represent early to late activation (all panels). **(C, D) Three block lines** and **3 gaps**. The overall isthmus boundary has an approximately hexagonal shape. Two of 6 possible re-entrant circuit morphologies are shown in panels **C and D**. **(E, F) Four block lines** and **4 gaps**. The overall isthmus boundary has an approximately octagonal shape. Two of the 14 possible circuit morphologies are shown in **E and F**. **(E)** Double-loop morphology. **(F)** Four-loop (quatrefoil) morphology.

From the previous description, it is evident that borders between source and sink are critical areas where functional conduction block can occur. Several re-entrant circuit configurations can arise from this process. The critical isthmus must have at least 1 entrance and 1 exit point so that the activation wavefront can travel through the isthmus, and at least 2 conduction block segments to separate the entrance and exit points. Various configurations of the re-entrant VT isthmus are depicted in Figure 6. In the circuit morphologies depicted in Figures 6A and 6B, there is a single entrance and a single exit point, with 2 segments of functional conduction block separating them. The difference between Figures 6A and 6B is of wavefront travel direction; both give rise to classic double-loop re-entrant circuits. The isthmus boundary shape is approximately rectangular, consisting of 2 segments for entrance/exit and 2 segments for conduction block. Polygonal shapes with a greater number of sides give rise to more entrance/exit points and conduction block segments. The isthmus boundary is approximately hexagonally shaped in Figures 6C and 6D, resulting in 3 entrance/exit points and 3 conduction block segments. Two of 6 possible circuit morphologies that can arise from this configuration are depicted in these panels. In each, there is a double-loop circuit. In Figure 6C, there are 2 entrance points, whereas in Figure 6D there are 2 exit points. Similarly, an isthmus boundary with an approximately octagonal shape gives rise to 4 entrance/exit points and 4 conduction block segments. Two of the 14 possible circuit morphologies that could arise from this configuration are depicted in Figures 6E and 6F. In Figure 6E, the entrance points are adjacent to each other and the exit points are adjacent to each other, giving rise to a double-loop re-entrant circuit. In Figure 6F, however, the entrance and exit points alternate, giving rise to a 4-loop (quatrefoil) re-entrant circuit, which has been observed in activation mapping 35, 36.

## Infarct Configuration Resulting in Isthmus Shape

**Figure 7:**
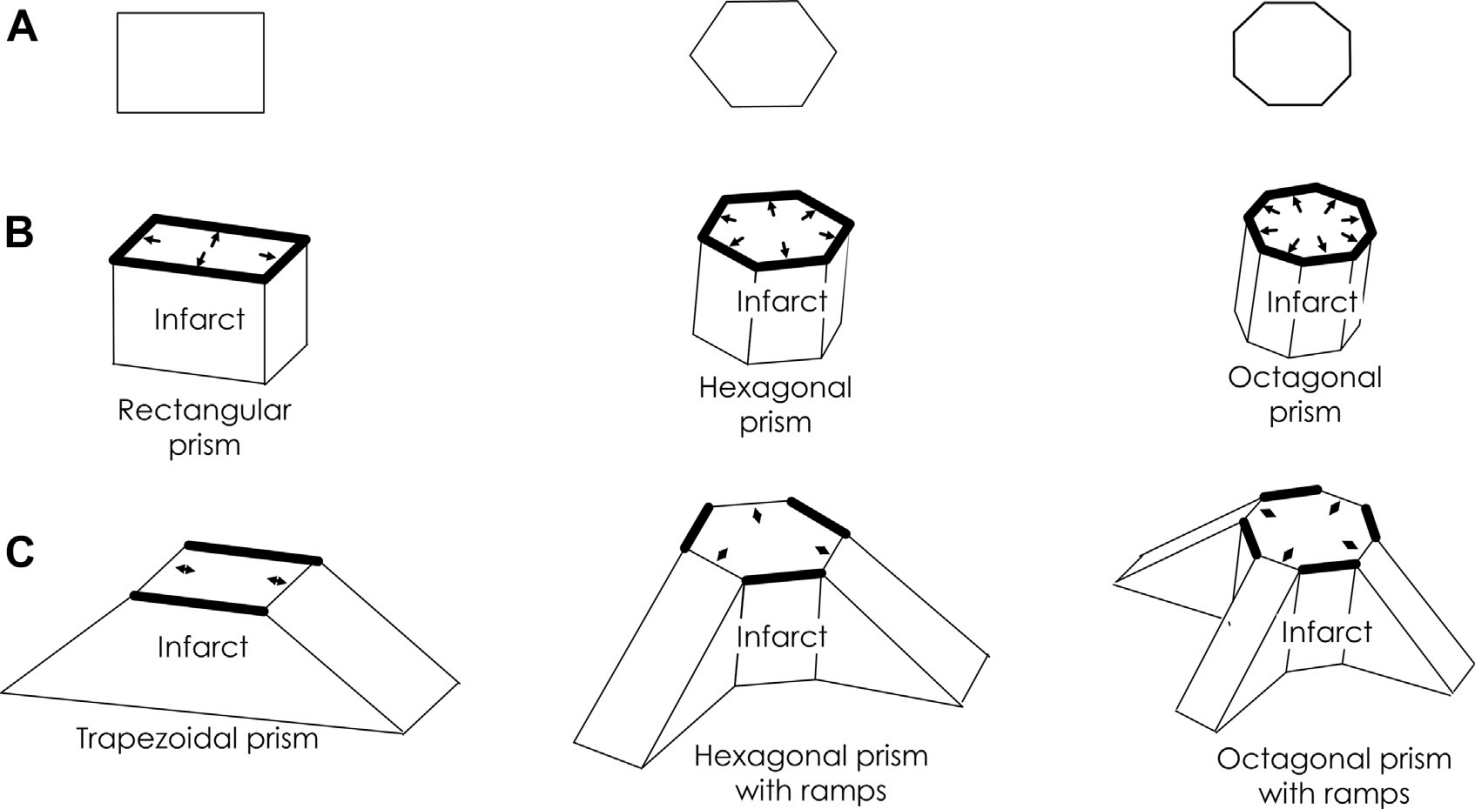
Geometric Models of Superficial Infarct Shape Related to the Critical Isthmus of the Re-Entrant Circuit In 2 dimensions, these shapes are rectangular, hexagonal, and octagonal **(A)**, as in Figure 6. In 3 dimensions, they form, respectively, a rectangular, hexagonal, and octagonal prism **(B)**. Suppose the myocardial surface is above each surface in **B**, then the thin infarct border zone is between the myocardial surface and the infarct surface. An activation wavefront traveling above the infarct would be blocked as it arrived at the infarct edges because of the large increase in myocardial thickness in the travel direction **(arrows and thick black lines)**. **(C)** As shown here, however, a more gradual change in thickness of the viable myocardium where the infarct changes as a ramp enables wavefront propagation out of the isthmus along these segments **(double-headed arrows)**.

The polygonal isthmus shapes depicted in Figure 6 can originate from alterations in IBZ thickness as described in Figures 4 and 5. In Figure 7A, the isthmus shapes of Figure 6 are repeated (rectangular, hexagonal, and octagonal). Suppose that the superficial infarct beneath the IBZ has the corresponding shape of the isthmus boundary in 3 dimensions, as depicted in Figure 7B. Let the IBZ be directly above each of the resulting prisms shown in Figure 7B. If an activation wavefront were to travel through any of these isthmus regions, it would be prevented from exiting outwardly across the isthmus boundary, owing to the sharp change to thicker tissue at the isthmus edges, resulting in critically convex wavefront curvature. These edges, where functional conduction block would occur all around, are depicted as thick black lines, and activation wavefront leading edges, noted as small arrows, will block there. Suppose however that along alternating segments of each polygonally shaped superficial infarct region, there is a gradual rather than steep transition to thicker viable tissue (Figure 7C). These are noted as shallow inclined ramps comprising portions of the superficial infarct in Figure 7C, with viable IBZ being adjacent to and above the superficial infarct regions. Thus, gradual changes to thicker IBZ occur along the shallow inclined ramps, enabling the exit of wavefronts from the isthmus at these segments without attaining a critically convex wavefront curvature (shown as double-headed arrows). At these edges, *ΔT* per unit distance *c* is relatively small, whereas, along the steeper edges of the superficial infarct, function block occurs as any wavefront tries to exit, noted by thick black lines in Figure 7C. Furthermore, wavefronts propagating through the outer pathway tend to gravitate toward the entrance points along the shallow ramps because of concave wavefront curvature in the inward direction and the ensuing speedup and coalescing of wavefronts in that direction. The superficial infarct configurations described in Figure 7C thus correspond to the 3 basic re-entrant circuit configurations depicted in Figure 6.

## Description of the Experimental Method Used for Source-Sink Measurements

Much of the work leading to the development of the source-sink or current-load mismatch model was investigated using in vivo post-infarction canine hearts. In total, experiments were done using 7 mongrel canines weighing 20 to 40 kg (26). For canine data, approval for conducting the experiments and analysis of the data was granted by the Institutional Animal Care and Use Committee of Columbia University Medical Center. The method for study of post-infarction canine hearts with activation mapping (6) and for the use of histologic analysis 30, 31 or magnetic resonance imaging (26) to determine the IBZ architecture, have been described in detail previously. Briefly, under pentobarbital anesthesia (15 to 30 mg/kg intravenously), the left anterior descending coronary artery was ligated near its base. The animals were allowed to recover, and 3 to 5 days post-infarction, they were prepared for electrophysiologic analysis (30). Under pentobarbital anesthesia, a 196- or 312-multielectrode grid with bipolar electrode configuration (with approximately 1 mm spacing between poles) was then sutured to the anterior left ventricle after opening the chest. The mapping system for acquisition and storage of the bipolar electrograms has also been described in detail elsewhere (37). Histologic analysis was done from samples obtained at 5-mm intervals in the X and Y directions throughout the IBZ in 4 ex vivo hearts. The resolution of the thickness measurements (Z axis) was 0.1 micron. Magnetic resonance imaging was done on 3 ex vivo post-infarction canine hearts, with a thickness resolution of 0.4 mm throughout the IBZ (26).

## Comparison of Activation Mapping to Source-Sink Model

**Figure 8:**
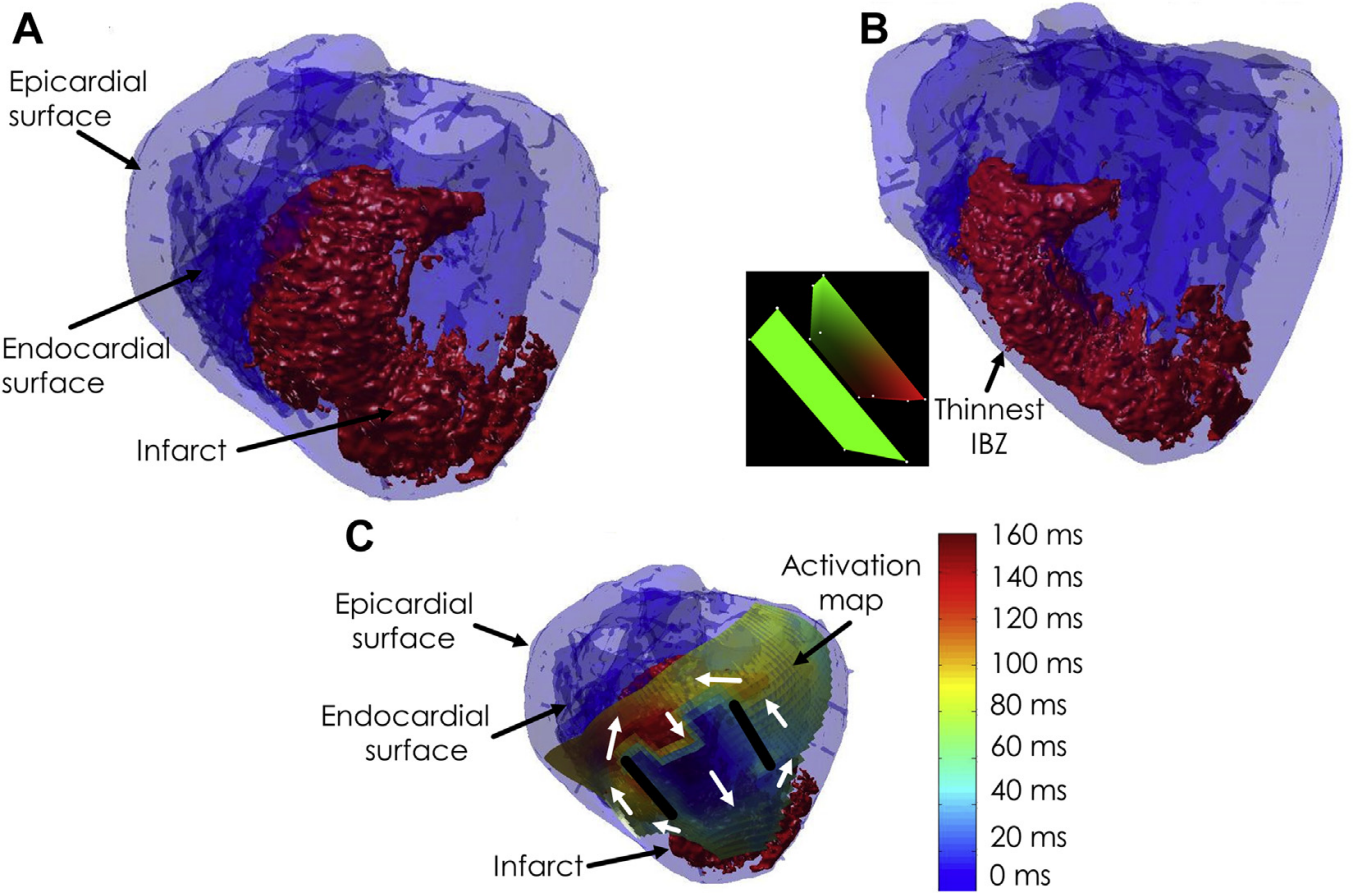
Endocardial and Epicardial Heart Surfaces With Infarct, Positioned by a Mathematical Registration Technique (38) **(A)** View from directly above the IBZ. **(B)** Side view with respect to IBZ. Inset shows orientation of a trapezoidal prism representing the infarct, with the heart surface depicted as a plane. **(C)** View from above the IBZ with overlaid activation map. **Color scale** provides activation times. Courtesy of Dr. Elliot R. McVeigh and Dr. Hiroshi Ashikaga, Johns Hopkins University. The **inset of B** was generated using map3d (39), as were Figures 9 and 10. IBZ = infarct border zone.

A complete rendering of an infarct, and the endocardial and epicardial surfaces of the left ventricle, with mathematical registration (38), are shown in Figure 8 for a post-infarction canine heart experiment. In the first panel, a view oriented from above the IBZ is provided. There is an evident sharp drop-off to thicker tissue at the lateral edges of the infarct. In Figure 8B, a view of the infarct is shown from the side. The IBZ is superficial to the infarct, toward the epicardial surface. Notice the thinness of the viable substrate there. Furthermore, there is a more gradual change to thicker IBZ away from the thinnest point, noted by an arrow. The infarct shape in 3 dimensions geometrically resembles the trapezoidal prism of Figure 7C (26), which is noted in the inset to the left of Figure 8B with the correct orientation, along with the epicardial surface (green plane in the inset). In the third panel, the activation map during VT is overlaid on the 3-dimensional heart and infarct rendering. In this map, early activation is noted in blue and late activation in red. A classic double-loop re-entrant circuit is present during VT, with the isthmus location precisely overlying the thinnest IBZ superficial to the infarct, and the lateral, functional block lines coinciding with areas of sharp drop-off in the infarct to thicker viable tissue, as would be expected from source-sink mismatch.

**Figure 9:**
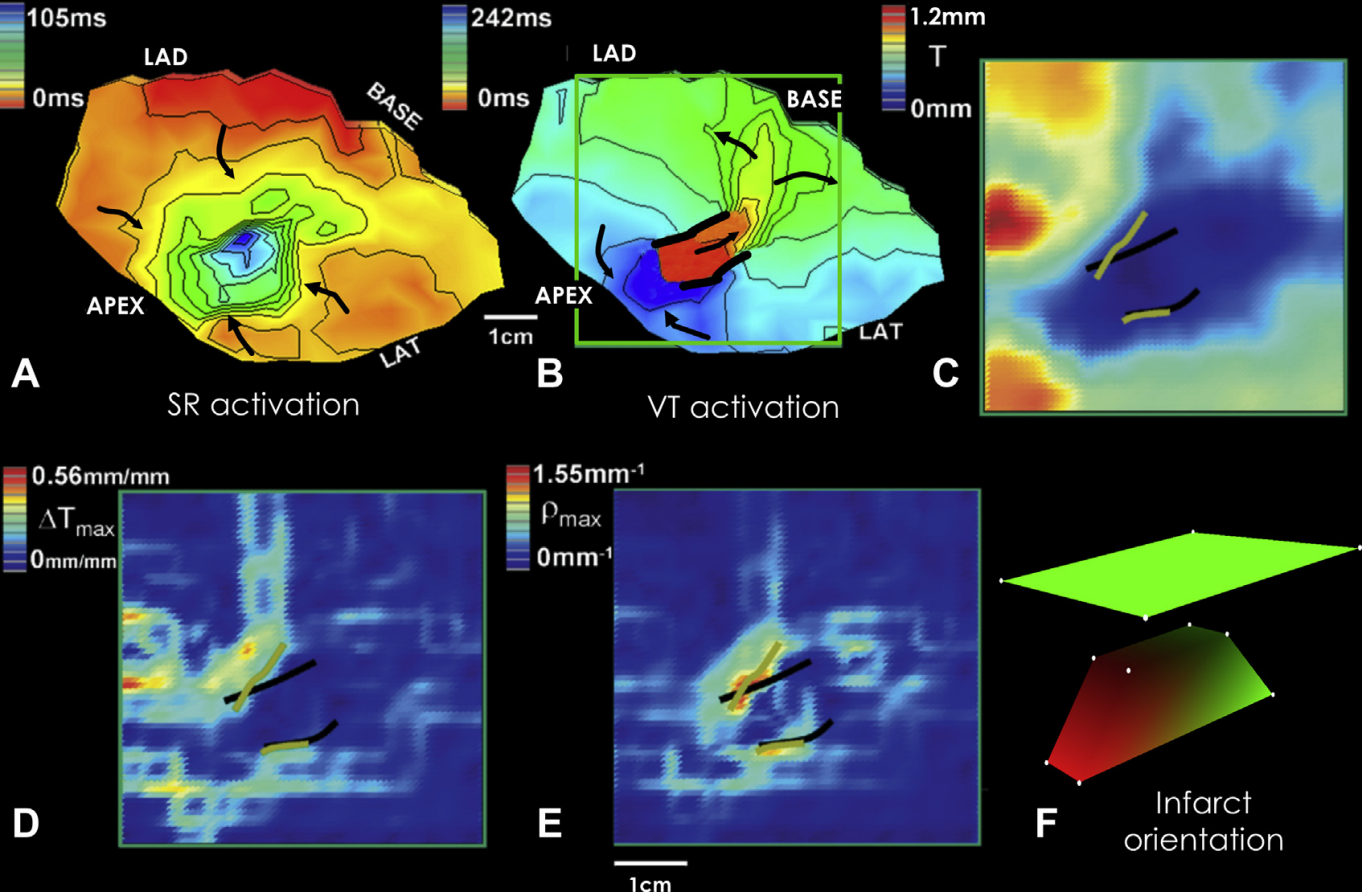
Correspondence of Activation Maps to Infarct Border Zone Thickness Activation maps **(A, B)**, and thickness maps determined by histologic analysis **(C to E)**. **(F)** Infarct shape as a trapezoidal prism, with the surface plane of the heart above, oriented to correspond to the other panels. Partially redrawn from Ciaccio et al. (26). LAD = left anterior descending artery; LAT = lateral; SR = sinus rhythm; VT = ventricular tachycardia.

In Figure 9, activation maps of sinus rhythm and re-entrant VT in the epicardial border zone of the anterior left ventricle are displayed in Figures 9A and 9B, respectively, for a post-infarction canine heart experiment. The bipolar electrode grid density for this experiment was 196 electrodes. Early activation is colored in red, with late activation shown in blue. During sinus rhythm, the activation wavefront tends to proceed from the grid edges toward the center. Unlike in Figure 3, the entire region activates; there are no fixed obstacles to the activation wavefront in this experiment. During VT, there is evident a classic double-loop re-entrant circuit (Figure 9B). The lateral, functional block lines are depicted as thick black lines. The electrical impulse propagates through the isthmus, exits, bifurcates, and then travels as 2 distinct wavefronts along the outer pathway, which then coalesce and re-enter the isthmus region, as noted by the black arrows. The thickness map *T* is shown in Figure 9C, with the thinnest IBZ colored dark blue and the thickest in red. This map was constructed from histologic analysis of the area outlined as a green square in the VT activation map of Figure 9B. The location of the functional block line locations from VT activation mapping are overlaid for reference as thick black lines (Figures 9C to 9E). The *ΔT* map is provided in panel D. Maximum *ΔT* was approximately 0.56 mm per millimeter spatial interval along the electrode grid, in accord with Figure 5B and Equation 3. The map of *ρ = ΔT/c·T* is presented in panel E, where *ρ* is the degree of wavefront curvature. It is listed as *ρ*_*max*_, which is the maximum absolute *ΔT/c·T* in the vector field at any particular point on the grid. At the maximum values of *ρ*_*max*_, colored red, orange, and yellow, estimated lines of block were drawn on the computerized grid in green. They approximately match the actual lines of block determined by activation mapping, overlaid for reference (thick black lines). The upper value of 1.55 mm^-1^ for *ρ*_*max*_ in panel E is in approximate agreement with the value of 2 mm^-1^ predicted by Equation 3. Thus source-sink mismatch provided a good estimate of actual functional block line location in this experiment.

Although the thickness map in Figure 9C is 2-dimensional, if the thickness of the superficial portion of the infarct were to be modeled as a geometric shape, it could be approximately represented as shown in Figure 9F. The superficial portion of the infarct is depicted as a trapezoidal prism, colored in red and green, and the heart surface as a plane colored in green. The plateau of the prism corresponds with that portion of the infarct directly beneath the isthmus region, which is the thinnest region of the border zone in Figure 9C. It is at this plateau that there is the shortest distance to the epicardial surface and therefore the thinnest viable tissue post-infarction. Thus, *T* is minimized at this location. At the lateral edges of the prism’s plateau, there are sharp changes to thicker tissue (i.e., *ΔT* is maximized). Thus, it is at these locations that *ρ = ΔT/c·T* is maximized and functional block can occur. Correspondingly, in Figure 9C, at thickness map *T*, there is a sharper change to other colors and thus sharper change in *T*, away from the lateral borders of the isthmus. Whereas, along the long axis of the prism in Figure 9F, there is a more gradual change to thicker tissue (areas with ramps in Figure 9F). This represents the more gradual change in thickness at the entrance and exit of the isthmus region, and a correspondingly more gradual change in color in Figure 9C, in correspondence to the isthmus long axis.

## Deduction of Isthmus Properties for Ablation

**Figure 10:**
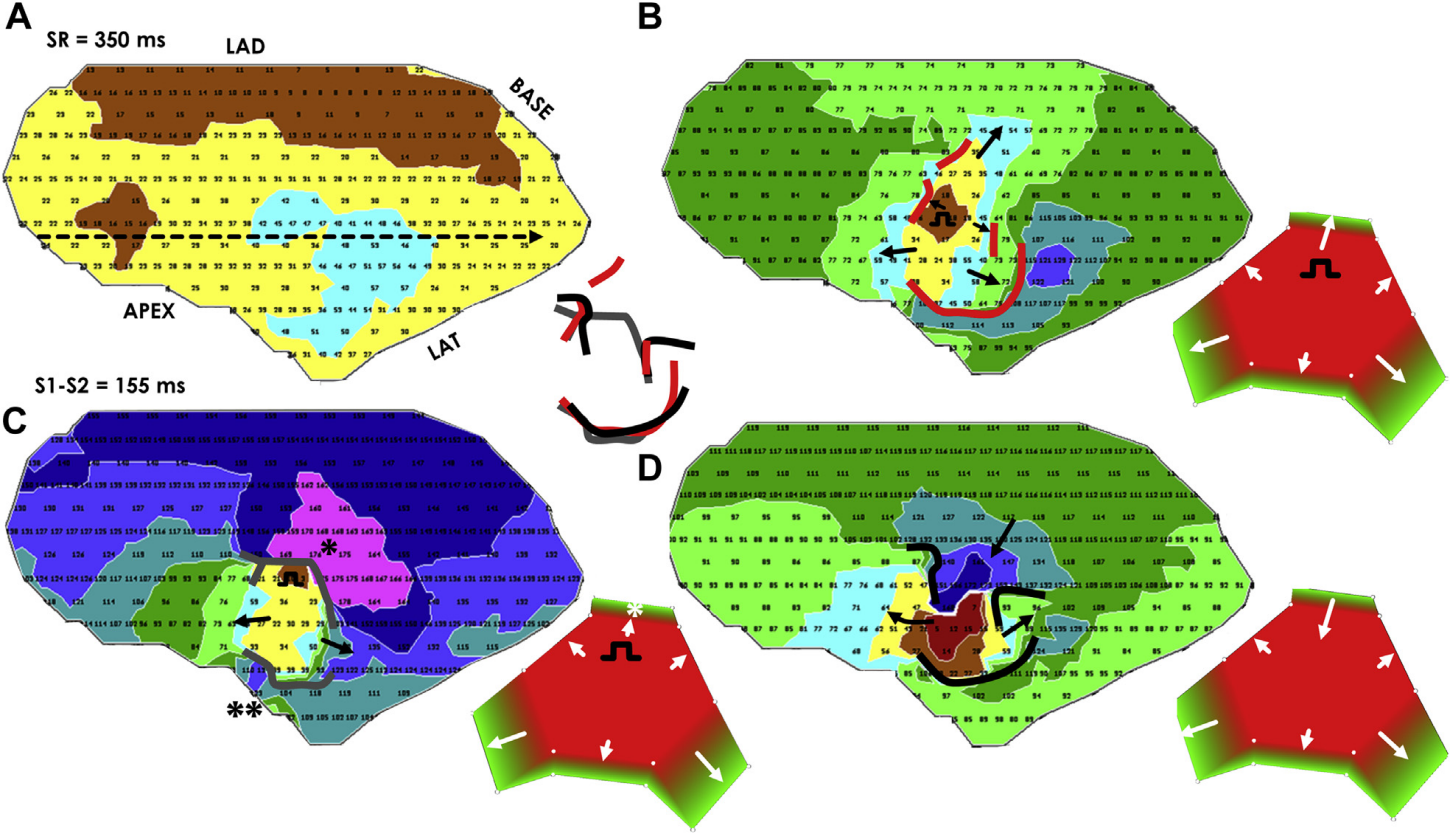
Premature Excitation Leading to Re-Entrant Ventricular Tachycardia With Stimulation Point Located Within the Re-Entry Isthmus Region Activation maps show **(A)** sinus rhythm and **(B)** S1-S1 stimulation, **(C)** S1-S2 (premature) stimulation, and **(D)** ventricular tachycardia. At center, functional block lines forming during S1-S1, S1-S2, and ventricular tachycardia are shown overlapped for correspondence. **(B to D, insets)** A geometric model of the superficial infarct shape, structured as a hexagonal prism with extending ramps. Conduction block occurs at **short right arrows** in these insets, whereas propagation of the wavefront proceeds at longer **white arrows** in the directions shown. Partially redrawn from Ciaccio et al. (41). In the **C** activation map and its **inset**, a single **asterisk** denotes the area where unidirectional block forms during premature stimulation. In the activation map of **C**, a **double asterisk** denotes the area where bidirectional block occurs during premature stimulation. LAD = left anterior descending artery; LAT = lateral; SR = sinus rhythm.

In this review, several mechanisms leading to the onset and maintenance of re-entrant VT and how these affect isthmus shape have been discussed. An ablation lesion should optimally be made within the isthmus confines to interrupt the circuit because it is a constrained region of approximately 2 cm across (34). Although magnetic resonance or other imaging, such as computed tomography (40), would be needed to detect wall thinning and to determine the infarct shape for source-sink modeling, based on the model itself, the electrophysiologic properties determined when a programmed electrical stimulus is applied within the confines of the isthmus boundary might also be useful to deduce the re-entry isthmus shape and thereby the best ablation lesion. This is shown as an example in Figure 10 for a post-infarction canine experiment. During sinus rhythm, electrical activation occurs throughout the IBZ (Figure 10A). When stimulating from within the isthmus with a coupling interval on the order of the VT cycle length (i.e., S1-S1) (Figure 10B), functional block forms at segments of the isthmus boundary (41). Applying a premature stimulus S1-S2, as in Figure 10C, it is possible to deduce the re-entrant circuit orientation, with the protoisthmus entrance being that gap segment at the isthmus border across which the activation wavefront traverses during the longer coupling interval of S1-S1, but not during the short coupling interval of S1-S2, because of source-sink mismatch leading to functional block. This entrance region is noted by the single asterisk in Figure 10C. Ablation across this so-determined boundary would be used to prevent re-entrant VT reinduction. The estimate is in good agreement with the actual configuration of the re-entry isthmus, shown in Figure 10D. The unidirectional block line noted by an asterisk in Figure 10C would overlie the isthmus entrance in Figure 10D, and could be ablated across to prevent reinduction of re-entrant VT. To show correspondence, the overlap of functional block lines from Figures 10B to 10D is delineated in the center panel of Figure 10.

Although no thickness data were obtained for the experiment depicted in Figure 10, the infarct configuration that would be expected from source-sink mismatch is shown in the lower right insets of Figures 10B to 10D. The isthmus shape is anticipated to be hexagonal, as in Figure 6C to 6D, and the 3-dimensional superficial infarct configuration would be expected to have the approximate shape of a hexagonal prism with ramps as shown in Figure 7 (lower center column). Gaps where propagation proceeds during S1-S1 are noted by long white arrows in the model configuration at lower right of Figure 10B. Functional block occurs only where there are segments with sharp increase in IBZ thickness in the outward direction (short white arrows). At the shorter coupling interval of S1-S2 (Figure 10C), functional block also occurs at the gap across the isthmus boundary closest to the stimulus site, which may be either due to its proximity and/or to a steeper *ΔT*. This would be the expected protoisthmus entrance location (white asterisk, Figure 10C inset). After the stimulus wavefront exits the 2 other gap segments, it then bifurcates around. There is insufficient time for recovery of excitability and re-entry to occur at 1 segment (**, 85 ms, Figure 10C), but there is sufficient time for recovery at the other segment (*, 173 ms, Figure 10C), leading to re-entry (Figure 10D). Propagation succeeds across all gap segments where lesser *ΔT* would be expected during re-entrant VT (Figure 10D inset).

**Figure 11:**
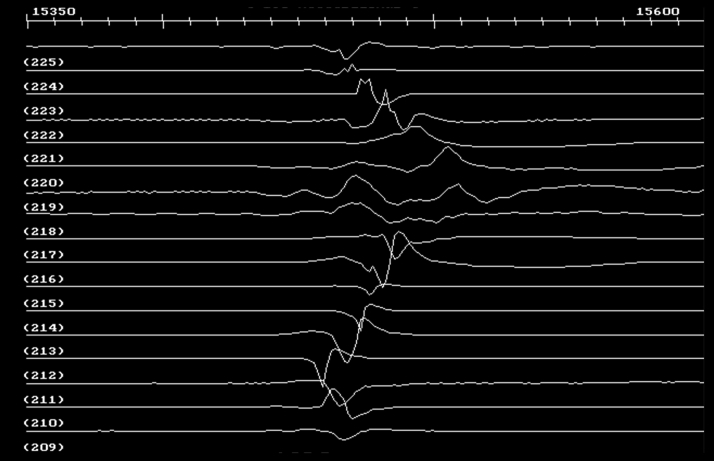
Electrograms Acquired During Sinus Rhythm for the Experiment Depicted in Figure 10 The channel number is given just below each trace; the interval shown is 250 ms. **Arrow** in Figure 10A shows sequence from tail to head (channels 209–225). Fractionation occurs where block lines form during stimulation and re-entrant VT, in accord with a previously described model of source-sink mismatch causing electrogram fractionation (41). Abbreviation as in Figure 9.

For completeness, examples of electrograms from the experiment of Figure 10A (sinus rhythm) at the level of the dashed arrow that is drawn, are shown in Figure 11. Electrogram channels from 209 to 225 correspond to the direction from tail to head of the arrow in Figure 10A. Fractionation occurs at areas where functional block lines form during S1-S1 and S1-S2 stimulation and VT (channels 218–221), in accord with a bipolar electrogram fractionation model described in detail previously (41).

**Figure 12:**
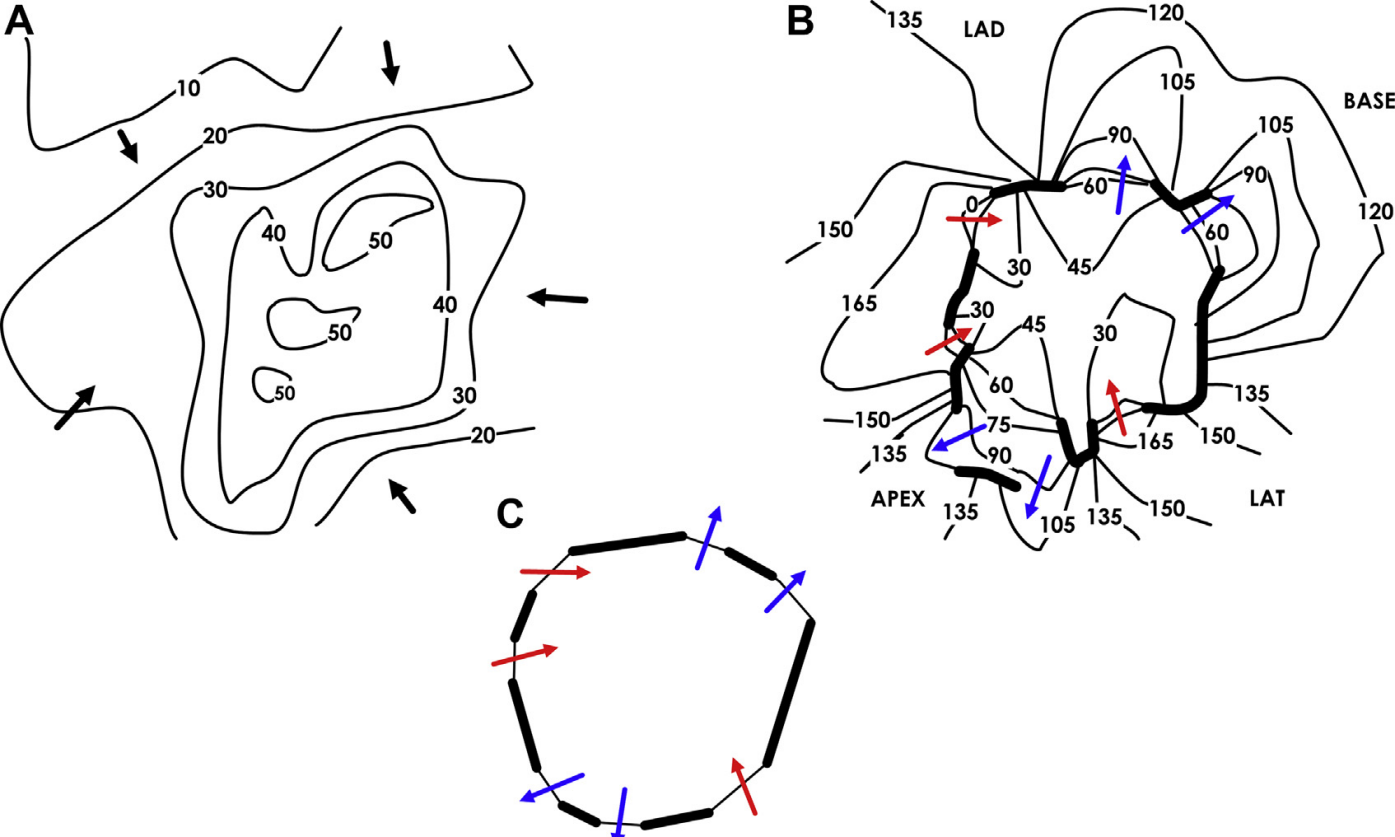
Four-Loop (Quatrefoil) Re-Entrant Circuit **(A)** Sinus rhythm activation map. **(B)** Ventricular tachycardia activation map. **(C)** The **isthmus shape** is approximately that of a tetradecagon (14-sided).

Finally, an example of 4-loop (quatrefoil) re-entry from a post-infarction canine experiment is shown in Figure 12. The sinus rhythm and VT activation maps are presented in Figures 12A and 12B, respectively. There is a degree of slowing, but not block, in proximity to the location of the isthmus boundary during sinus rhythm. During VT, 1 of the entrances and 2 of the exits have dual areas of propagation separated by lines of functional block. Overall, however, there is an alternation between the 2 combined entranceways and the 2 combined exits, generating a 4-loop re-entrant circuit. Based on combined entrance/exit points, the isthmus boundary shape is approximately a tetradecagon (14-sided), as noted in Figure 12C.

Partially redrawn from Ciaccio et al. (36). LAD = left anterior descending artery.

## Expected Circuit Morphologies

In canine post-infarction, it is rare for both re-entrant circuit morphologies to be inducible in the 4-sided isthmus boundary case (Figures 6A and B). Moreover, regardless of the polygonality of the isthmus boundary, 2 or at most 3 circuit morphologies are typically observed to be inducible at any 1 location 15, 16. Thus, not all potential circuit morphologies are actually realized. For a unidirectional block line to form during premature stimulation at the protoisthmus entrance, the value of *ρ* must be sufficiently large to cause critically convex curvature at the S1-S2 coupling interval (32). If *ΔT* is insufficiently steep, *ρ* will not be large, and unidirectional block will be unlikely to occur. Even at a candidate protoisthmus entrance segment where block does occur during S1-S2, if the segment length is too short, the wavefront may sweep around too rapidly and block bidirectionally prior to recovery of excitability on the stimulus side, again preventing re-entry. Anisotropy will also influence the arrival time at the opposite side and can result in insufficient delay. Furthermore, if *ρ* is too large at the protoisthmus exit, even though there is a delay in arrival so that the coupling interval for reactivation is on the order of the VT cycle length, functional block may occur there, again preventing VT induction. Thus, whether a particular re-entrant circuit morphology actually occurs is dependent on the particulars of the source-sink boundary properties and anisotropy, which is the subject of further study.

## Other Mechanisms Contributing to Functional Block

At locations of source-sink mismatch bounding the re-entry isthmus, differences in molecular-level properties of the substrate and gap junctional connections between cells may also contribute to the formation of functional block 7, 42. Furthermore, when the re-entry isthmus is oriented with its long axis in parallel with myocardial fibers, hindrance of lateral conduction can in part be caused by anisotropy (43). Activation wavefront propagation may markedly slow transverse to muscle fibers resulting from a paucity of operative gap junctional connections along this orientation. Block would occur if the oncoming wavefronts propagating along the outer pathways interrupt travel of the very slow, laterally conducted wavefronts at the isthmus boundary. Yet, this would not explain lateral conduction block when the isthmus long-axis is not precisely in parallel with myocardial fibers, as is often the case (34), and it would not explain block at all segments of the isthmus boundary when its shape is polygonal with more than 4 sides, some or all of which would not be aligned with muscle fibers. In cases in which the surviving myocardial layer at the thinnest IBZ is very tenuous, on the order of tens of microns thick, discontinuous clusters of cardiomyocytes may result, which could potentially lead to conduction block within the isthmus itself. It has been shown in swine post-infarction, however, that accumulation of myofibroblasts there can serve to preserve electrical continuity (44) so that the critical isthmus still conducts during premature stimulation.

## Comparison of Canine With Clinical Re-Entrant Circuits

The source-sink model described in this and prior studies is mostly based upon experiments done with canine post-infarction VT 5, 26, 32, 33. Although there are differences in the electrophysiology of canine versus clinical myocardial substrate when re-entrant VT is inducible, there are also many similarities. These include the presence of an IBZ, which is typically subepicardial in canine and subendocardial in clinical cases, the presence of a nonconducting infarct region at depth, the possibility of induction of a re-entrant circuit via programmed electrical stimulation from the IBZ, formation of a unidirectional block line, and activation wavefront bifurcation around the line followed by coalescence and re-entry into the previously excited region, typically resulting in the formation of a double-loop re-entrant circuit that drives VT, with functional block bounding the lateral borders of the isthmus. The average dimensions of the re-entry isthmus is similar in canine versus human post-infarction 34, 45; however, a larger pool of experiments should be used to validate the findings summarized in this review. Furthermore, histologic analysis cannot be used for thickness mapping in clinical patients; thus, magnetic resonance or other imaging would be required, but this may be problematic in patients with an implantable cardioverter defibrillator or other device. Imaging methods and implantable devices are being developed to reduce or eliminate the difficulties with obtaining IBZ architectural information for input to the source-sink model. The post-infarction canine experiments discussed in this review were analyzed 3 to 5 days after left anterior descending artery ligation. Healed infarcts in canine hearts may have altered electrophysiologic properties, and require further investigation.

## Conclusions and Outlook

In this work, activation mapping in the post-infarction IBZ was reviewed to show the predictive capability of the source-sink mismatch model for localizing functional conduction block leading to the onset and maintenance of re-entrant VT. An overview is provided in Central Illustration. Emphasis was placed on the shape of the superficial infarct in the subepicardium (canine) or subendocardium (human patients). Extending the model, it is apparent that functional block at areas of source-sink mismatch can be caused by any alterations in the geometry of the viable electrically conducting medium in myocardial tissue. These alterations in geometry can be due, for example, to presence of an infarct region, and would then coincide with an IBZ as described here, but could also be due to fibrosis or to discontinuity of muscle fiber bundles. Induction of VT, maintenance of the re-entrant circuit, and presence of limited numbers of morphologies that are inducible from constrained stimulus locations are accounted for by the source-sink model. The presence of functional block in intramural re-entrant circuits causing VT can also be explained by the same mechanism (33).

**Central Illustration:**
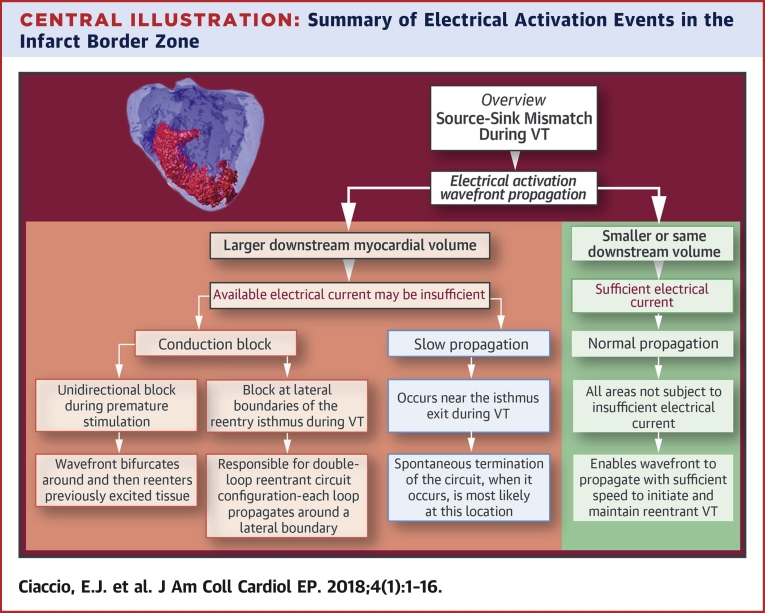
Summary of Electrical Activation Events in the Infarct Border Zone The figure provides an overview of source-sink mismatch as it applies to re-entrant ventricular tachycardia. Whether or not the activation wavefront will propagate within the infarct border zone region depends upon the availability of electrical current for downstream activation of the viable myocardial substrate. When the downstream volume (the sink) is of lesser or equal size as compared with the previously activating tissue (the source), there will be sufficient electrical current for activation **(right column in the figure)**, which is applicable to most of the infarct border zone. However when the sink is substantially larger in size as compared with the source, the current available for activation downstream is likely to be insufficient. Slow conduction or block will result (left 3 columns in the figure) which are crucial components of the re-entrant ventricular tachycardia circuit and the double-loop configuration. VT = ventricular tachycardia.

Once arcs of functional block are localized based upon the geometry of the viable substrate via magnetic resonance, computed tomography, or other imaging, it would be possible to ablate these arrhythmogenic regions during electrophysiologic study, thereby preventing re-entrant VT induction, without the need to induce arrhythmia and subsequently map the activation pattern. Ablating across a channel so formed, where the electrical impulse is constrained, would minimize lesion size. The model might also be applied to atrial fibrillation substrate, particularly when obstructions caused by fibrosis and discontinuity of muscle fiber bundles, substrate for source-sink mismatch, are present. Based upon this work, the answer to the question “Are structure and function distinguishable concepts in cardiac electrophysiology?” would be the affirmative: structure indicates function.
